# Determining the potential link of self-compassion with eating pathology and body image among women: a longitudinal mediational study

**DOI:** 10.1007/s40519-021-01144-1

**Published:** 2021-02-18

**Authors:** Fidan Turk, Stephen Kellett, Glenn Waller

**Affiliations:** Department of Psychology, University of Sheffield, Cathedral Court, 1 Vicar Lane, Sheffield, S1 2LT UK

**Keywords:** Eating pathology, Self-compassion, Body image, Longitudinal analysis, Mediator, Shame

## Abstract

**Purpose:**

This longitudinal study aims to determine what factors mediate the previously established link between self-compassion and eating pathology/body image concerns, over a 6-month period.

**Methods:**

A community sample of 274 adult women (*M* = 29.50 years) completed standardised validated measures of self-compassion (Self-Compassion Scale), rumination (Ruminative Thought Style Questionnaire), shame (Other as Shamer Scale), perfectionism (Short Form of the Revised Almost Perfect Scale), self-criticism (Levels of Self-Criticism Scale), eating pathology (Eating Disorder Examination Questionnaire) and body image (Body Shape Questionnaire). They reported levels of: self-compassion at Time 1, potential mediators (rumination, shame, self-criticism, perfectionism) at 3 months; and eating pathology and body dissatisfaction a further 3 months later. Missing data were handled using multiple imputation. Stepwise multiple regression showed that shame was the most consistent mediator.

**Results:**

Shame acted as a full mediator of the self-compassion-eating/body image relationship {respectively, [B = .04, SE = .01, t(268) = 3.93, *p* < .001], [B = .33, SE = .15, t(268) = 2.25, *p* < .05]}. Discrepancy perfectionism also played a mediating role in the link between self-compassion and body image dissatisfaction [B = .59, SE = .28, t(268) = 2.10, *p* < .05].

**Conclusion:**

These results support the hypothesis that self-compassion is relevant to eating pathology and body image disturbance, and demonstrate that shame is an important mechanism in that relationship. This pattern suggests that interventions that reduce shame should be considered when addressing issues relating to self-compassion and its links to eating disorders.

**Level of evidence:**

Level IV, multiple time series without intervention.

## Introduction

Eating disorders are serious mental health conditions characterized by a persistent course, comorbidity with other psychiatric conditions, medical issues, and high mortality rates [1]. Body image concerns and eating pathology, including dysfunctional eating attitudes (e.g., giving too much time and thought to food and appearance) and behaviours (e.g., binging and purging), are core to the maintenance of eating disorders [2]. However, such eating pathology and body issues are common in the non-clinical population [3]. Epidemiological studies report that eating disorders are highly prevalent worldwide, especially in women from Western and westernized countries [4]. Therefore, this study addresses body image and eating pathology in a non-clinical group of young adult women, who are at risk of developing such problems [5]. The background of these women was that they were recruited in the United Kingdom, which is a relatively typical Western country in terms of levels of eating pathology, and can therefore be considered to be representative of a substantial proportion of the world’s population.

Recently, self-compassion has been suggested as a process that can reduce such eating pathology and body image issues. Self-compassion can be defined as being mindful, understanding, and nurturing toward the self during challenging and suffering times [6]. It involves three components: self-kindness (vs. self-judgment); common humanity (vs. isolation); and mindfulness (vs. over-identification) [6]. A recent meta-analysis has suggested that self-compassion is relevant to eating pathology and body dissatisfaction, and that addressing self-compassion can be effective therapeutically, with medium effect sizes [7].

Despite this evidence of an association between self-compassion and eating pathology/body dissatisfaction, the potential mechanism underpinning that link is unclear. In particular, it is not known how self-compassion leads to positive changes in eating pathology and body image issues. Greater understanding of such processes can enhance the outcome of interventions by identifying their effective components and targeting them for intervention [8]. Thus, this research considers the potential role of self-compassion in understanding the mechanisms underlying eating pathology and body image dissatisfaction, particularly in mediational models [9].

Most research on such mechanisms has utilized cross-sectional designs, in which self-compassion and its correlates have been investigated at a single time point [10, 11]. However, cross-sectional study designs do not truly test the temporal precedence of change. Therefore, such studies fail to represent true mediational processes, which necessarily develop over time [12]. Therefore, this study will address the potential role of mediators in the relationship between self-compassion and eating pathology/body dissatisfaction, using a longitudinal design.

Four potential mediators are proposed—perfectionism, self-criticism, rumination and shame. Each of these has been shown to be associated with self-compassion and eating pathology/body dissatisfaction [11–18]. For instance, individuals with a lack of self-compassion are likely to be less forgiving of their errors, and hence more perfectionist. Those individuals might be more likely to engage in eating disorder behaviours as an attempt to control their image to try to achieve the ‘perfect’ idealized physical body. In contrast, self-compassionate people are prone to be less self-judgemental, making them less self-critical. Hence, even if they do not meet an ‘ideal’ body image, they are less likely to have eating pathology. Similarly, they are less likely to feel shame following a perceived failure (e.g., not having an ideal body), which makes them less likely to engage in eating pathology behaviours to avoid that feeling of shame [10]. Regarding rumination, individuals with increased self-compassion are likely to be emotionally more open, and hence less likely to suppress their emotions and ruminate. Therefore, they are less prone to engage in eating pathology behaviours to regulate their negative emotions [19].

Despite the growing literature on these issues, theoretical models are still unclear as to whether self-compassion or these proposed mediating variables should have the lead causal role as independent variable. Consequently, a case could be made for a number of studies, each investigating different temporal models. However, in this case self-compassion is considered as the primary variable in the proposed causal chain, because it can be argued that the early caregiving environment (e.g., parental warmth, kindness) is likely to lead to self-compassion developing earlier than features such as perfectionism, self-criticism, rumination and shame [20].

**Fig. 1:**
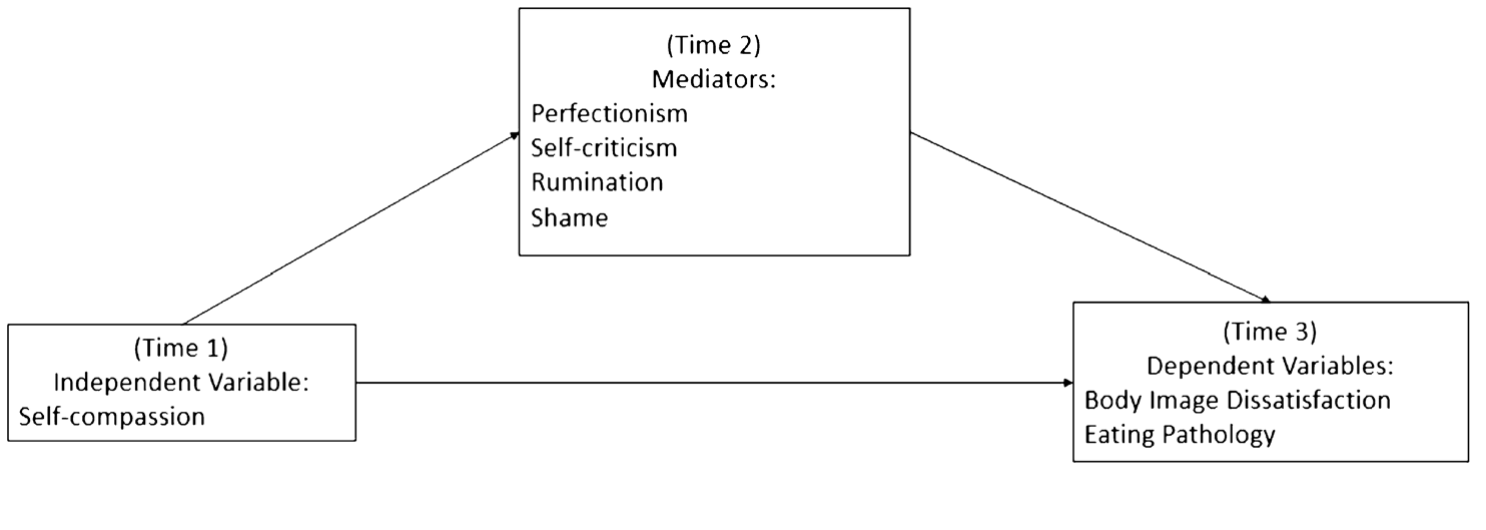
Proposed mediational model, which suggests that the longitudinal relationship between self-compassion and eating pathology/body image will be mediated by perfectionism, self-criticism, rumination, and shame

The aim of the current study is to examine longitudinally the mechanisms linking self-compassion with eating pathology and body image issues among women. Perfectionism, self-criticism, rumination and shame were considered as potential mediators in those relationships (Fig. 1). As outlined above, we hypothesized longitudinal links between:

1. self-compassion (independent variable) and eating pathology/body image (dependent variables);
2. self-compassion and perfectionism/self-criticism/rumination/shame (mediators);
3. perfectionism/self-criticism/rumination/shame (mediators) and eating pathology/body image (dependent variables).

Finally, we hypothesized that the longitudinal relationship between self-compassion and eating pathology/body image would be mediated by perfectionism, self-criticism, rumination, and shame.

## Method

### Ethical issues

Ethical approval for the research study was obtained from the Department of Psychology Ethics Committee at the University of Sheffield (reference 031350). The study was pre-registered with ASPREDICTED (no: 32863).

### Design

This mediational study employed a longitudinal design over 6 months. Data were collected at three time points to reflect the three steps in the hypothesised model. We collected the levels of: self-compassion at Time 1; potential mediators (rumination, shame, self-criticism, perfectionism) at 3 months (Time 2); and eating pathology and body image dissatisfaction a further 3 months later (Time 3).

### Participants

An a priori sample size calculation was undertaken using G*power 3.1.9.4 [21] and Cohen’s (1992) table [22]. These indicated that 97 participants were required in the third and final stage (80% power; *p* < 0.05, assuming a medium effect size, as shown in the literature [7]. Assuming a conservative 30% attrition rate at each stage, we aimed to recruit at least 200 women at Time 1.

The eligibility criteria were that participants needed to be female, 18 + years old, and fluent in English. If they were male, under 18 years old, not fluent in English, or diagnosed with a psychotic/neurological condition, they were directed out of the survey (*N* = 102). We only included English speakers to avoid risks of translation issues adding uncontrolled variability to the data. Of the 274 women who consented and participated at baseline (Time 1), 184 (67.1%) completed at Time 2 (3 months post-baseline), and 169 (61.7%) completed the final stage (Time 3 – 6 months post-baseline. Participants still could complete stage 3 if they had not completed stage 2 (*N* = 90). To summarise, the attrition rate from time 1 to time 2 was 32.9%, and it was 38.3% from time 1 to time 3. The Time 3 N indicated that the study was still appropriately powered (see above).

Participants’ ages ranged from 18 to 70 years (*M* = 29.50 years, *SD* = 9.09). They had a range of academic experience (22.6% high school, 36.9% Bachelor’s degree, 28.1% Master’s degree, and 12.4% doctoral degree). They self-identified as belonging to the following ethnic/racial groups: 62% white; 13.5% mixed/multiple ethnic groups; 10.2% South Asian/Asian British; 4% Black/African/Caribbean/Black British; and 10.2% other. They had a range of employment statuses (43.4% employed, 48.2% students, 4.7% self-employed).

### Measures

Using Qualtrics software, the participants completed measures of demographic characteristics (age, gender, education level, and ethnicity). Participants self-reported weight and height (used to calculate body mass index [BMI]) and completed self-report measures of: self-compassion; perfectionism; self-criticism; rumination; shame; body image; and eating pathology.

#### Independent variable (Time 1)

Self-compassion was measured using the Self-Compassion Scale (SCS) [23]. This 26-item scale is divided into the following six subscales: self-kindness, self-judgment, common humanity, isolation, mindfulness, and over-identification. The SCS shows good validity and internal consistency (*α* = 0.91 for self-compassion and *α* = 0.89 for self-judgment) [24].The self-compassion scale has shown good test–retest reliability over three weeks in past research, with test–retest correlation of 0.93 [24]. To determine the overall self-compassion score, first, the negative items were reversed, and then all six subscale means were computed.

#### Mediators (Time 2)

*Perfectionism* was assessed using the Short Form of the Revised Almost Perfect Scale (SAPS) [25]. The SAPS addresses two dimensions of perfectionism—standard (high performance expectations) and discrepancy (self-critical performance evaluations). The items are rated on a 7-point Likert scale ranging from 1 = Strongly Disagree to 7 = Strongly Agree. Responses were summed, with higher scores reflecting greater levels of each type of perfectionism. *Self-criticism* was measured with the Levels of Self-Criticism Scale (LOSC) [26]. The LOSC assessesed comparative self-criticism (CSC—a negative view of the self in comparisons with others) and internalized self-criticism (ISC—a negative view of the self in comparison with internal, personal standards). The SCS has good internal consistency (*α* = 0.84) and ISC (*α* = 88). Respondents rated items on a 7-point Likert scale that ranged from 1 = Strongly Disagree to 7 = Strongly Agree. Responses were summed, and higher scores reflected greater self-criticism. *Rumination* was assessed with the Ruminative Thought Style Questionnaire (RTSQ) [27]. Internal reliability for the RSTQ is excellent (*α* = 0.92), and it has strong convergent and divergent validity [27]. All responses are recorded on a 7-point Likert scale from 1 = Not at all to 7 = Very well. Item scores were summed, with higher scores indicating greater rumination. *Shame* was examined with the Other as Shamer Scale (OAS) [28]. The OAS has been shown to have a high α level (0.96) [28]. It also has good test–retest reliability (intraclass correlation coefficient = 0.97) [29]. Participants respond on a 5-point scale that ranges from 0 (never) to 4 (almost always). Higher summed scores reflect greater shame.

#### Dependent variables (Time 3)

*Body image* was measured with the shortened form of the Body Shape Questionnaire (BSQ-16) [30]. The BSQ-16 is a self-report measure of body dissatisfaction. BSQ alphas range from 0.93 to 0.96, and it has good concurrent and discriminant validity [30]. It demonstrates good test–retest reliability [31]. Participants respond from 1 (never) to 6 (always) for each item. Items are summed, and higher scores indicate greater body image dissatisfaction. *Eating pathology* was examined with a subset of scales from the Eating Disorder Examination Questionnaire—version 6.0 (EDE-Q) [32]. The EDE-Q has good validity and reliability [33]. Results indicate excellent 2-week test–retest reliability for the four subscales of the EDE-Q [34]. Only the Restraint and the Eating Concern subscales were used to measure eating pathology, as they do not reflect body image concerns (which were measured by the BSQ-16). EDE-Q items are rated on a seven-point scale, ranging from 0 (no days/not at all) to 6 (everyday/markedly). For each subscale, the responses for the relevant items were added together and that sum was divided by the total of items forming the subscale. Then, to obtain the overall EDE-Q score, the two subscale scores were summed and divided by two. Higher scores indicate greater eating pathology.

### Procedure

Recruitment of the sample was conducted through multiple sources, including leaflets displayed within a university, internet adverts including mail groups, and social media. Prior to any data collection, informed consent was obtained from all participants. The current study used the participants from a previous cross-sectional study (Turk, Kellett, Waller, under review) for the baseline (Time 1) data. Women from that study were asked to provide their email address, if they were willing to complete further questionnaires. 274 participants from 369 agreed to complete the longitudinal study.

### Data analysis

Binary logistic regression analyses were used to determine whether there were any significant differences in Time 1 scores between those who did or did not participate at stages 2 and 3. To reduce the risk of such attrition influencing outcomes, multiple imputations (20 imputations) were used to correct for missing data. The hypotheses were tested using Pearson correlations to determine bivariate association. To test mediation, the Baron and Kenny method was used, as it is compatible with multiple imputation datasets [35]. Three simple regressions were used to address hypotheses 1–3, and then a stepwise multiple regression analysis was used to test hypothesis 4.

## Results

### Attrition analysis

There were 11 variables at time 1 (self-compassion, rumination, shame, standard perfectionism, discrepancy perfectionism, internalized self-criticism, comparative self-criticism, eating pathology, body image dissatisfaction, age and BMI) that might have affected whether participants dropped out or not. These were compared across those participants who did or did not take part at times 2 and 3. Binary logistic analysis shows that there was a significant difference between who participated and those who did not at time 2 (*χ*^2^ = 20.66, df = 11; *p* < 0.05). The only variable at time 1 that was associated with attrition at time 2 was higher EDE-Q scores, which were associated with higher drop-out (*p* < 0.05). There was no significant difference in time 1 scores between those who participated at time 3 and those who did not (*χ*^2^ = 13.46, df = 11; *p* = 0.26).

### Descriptive statistics

Table 1 presents the descriptive statistics for the study variables. Scores on the SCS, RTSQ, and BSQ-16 were similar to those established for other non-clinical populations. However, scores on the LOSC, SAPS, and EDE-Q were slightly higher than previous studies (Table 1).

**Table 1:** Descriptive statistics and internal consistency of the questionnaires, with means compared to those of other studies with comparable populations (Pooled results from 20 imputations, *N* = 274)

	Present study M (SD)	*α*	Previous studies M (SD)
Time 1-self-compassion (SCS)	2.89 (*0.68*)	0.76	2.99 (*0.61*) [38]
Time 2-shame (OAS)	24.46 (*17.5*)	0.94	20.0 (*10.1*) [28]
Time 2-rumination (RTSQ)F	93.01 (*31.6*)	0.94	88.94 (*17.78*) [27]
Time 2-standard perfectionism (SAPS)	22.79 (*4.97*)	0.84	24.12 (*3.63*) [25]
Time 2-discrepancy perfectionism (SAPS)	18.75 (*6.95*)	0.89	14.2 (*3.31*) [25]
Time 2- comparative self-criticism (LOSC)	45.04 (*14.23*)	0.81	35.99 (*9.99*) [36]
Time 2-internalized self-criticism (LOSC)	48.95 (*12.25*)	0.90	35.82 (*11.84*) [36]
Time 3-body image (BSQ-16)	46.65 (*24.83*)	0.94	51.36 (*17.67*) [39]
Time 3-eating pathology (EDE-Q)	1.97 (*1.99*)	0.95	1.55 (*1.20*) [37]

*SCS* Self-Compassion Scale, *OAS* Other as Shamer Scale, *RTSQ* Ruminative Thought Style Questionnaire, *SAPS* Short Form of the Revised Almost Perfect Scale, *LOSC* Levels of Self-Criticism Scale, *BSQ-16* Shortened form of the Body Shape Questionnaire, *EDE-Q* Eating Disorder Examination Questionnaire

It was not possible to calculate Cronbach’s alpha values from multiple imputation data, so completer data were used. Scores from community sample studies

### Bivariate correlations

Table 2 presents the correlations among the proposed mediators, predictor and outcome variables. All relationships were significant except for standard perfectionism, which was only significantly correlated with discrepancy perfectionism and internalized self-criticism. Shame was very strongly correlated with comparative and internalized self-criticism (0.78 and 0.60). Therefore, to reduce the risk of multicollinearity due to the use of variables that measure the same construct, comparative and internalised self-criticism were omitted from the final analyses.

**Table 2:** Correlations between hypothesised independent, mediating and dependent variables, prior to running the mediator analyses (Pooled results from 20 imputations, *N* = 274)

	1	2	3	4	5	6	7	8	9
1. T1-self-compassion	–								
2. T2-standard perfectionism	− 0.03	–							
3. T2-discrepancy Perfectionism	− 0.55**	0.20**	–						
4. T2-rumination	− 0.40**	0.10	0.48**	–					
5. T2-shame2	− .58**	− 0.01	0.47**	0.54**	–				
6. T2- comparative self-criticism	− 0.61**	0.00	0.53**	0.52**	0.78**	–			
7. T2- internalized self-criticism	− 0.63**	0.31**	0.62**	0.53**	0.60**	0.65**	–		
8. T3-body image	− 0.39**	0.11	0.42**	0.39**	0.46**	0.50**	0.47**	–	
9. T3- eating pathology	− 0.28**	0.02	0.25**	0.27**	0.44**	0.39**	0.35**	0.76**	–

*T1* Time 1, *T2* Time 2, *T3* Time 3; ** < 0.01

### Mediation analysis

#### Eating pathology

##### Hypothesis 1

Higher levels of self-compassion were associated with eating pathology over the full period of 6 months [*B* = −0.54, *SE* = 0.14, *t*(272) = 4.04, *p* < 0.001]. To rule out the possibility that a link between self-compassion and eating pathology might be explained by weight, the association of self-compassion with BMI was tested. It was shown that BMI was not associated with self-compassion (*r* =  − 0.05, *p* = 0.44).

##### Hypothesis 2

Higher levels of self-compassion at time 1 were associated with lower levels at time 2 of discrepancy perfectionism [*B* =  − 4.68, *SE* = 0.56, *t*(272) = 8.41, *p* < 0.001]; rumination [*B* =  − 14.67, *SE* = 2.67, *t*(272) = 5.49, *p* < 0.001]; shame [*B* =  − 12.07, *SE* = 1.32, *t*(272) = 9.12, *p* < 0.001]. However, self-compassion was not related to standard perfectionism [*B* =  − 0.16, *SE* = 0.40, *t*(272) = 0.41, *p* = 0.68].

##### Hypothesis 3

At time 2, only higher levels of shame significantly predicted greater eating pathology at time 3 [*B* = 0.04, *SE* = 0.01, *t*(269) = 4.101, *p* < 0.001]. The remaining time two potential mediators were not related to eating pathology (standard perfectionism [*B* =  − 0.05, *SE* = 0.03, *t*(269) = 0.20, *p* = 0.84]; discrepancy perfectionism [B = 0.01, *SE* = 0.02, *t*(269) = 0.55, *p* = 0.58]; rumination [*B* = 0.01, *SE* = 0.01, *t*(269) = 0.18, *p* = 0.86]).

##### Hypothesis 4

In the final mediational stage in the analysis, self-compassion [*B* =  − 0.03, *SE* = 0.17, *t*(268) = 0.20, *p* = 0.84] was no longer a significant predictor of eating pathology after controlling for the mediating variables. Of the four potential mediators, only shame played a significant role [*B* = 0.04, *SE* = 0.01, *t*(268) = 3.93, *p* < 0.001] (Table 3). The remainder did not mediate the self-compassion-eating pathology link (standard perfectionism [B =  − 0.01, *SE* = 0.03, *t*(268) = 0.19, *p* = 0.77]; discrepancy perfectionism [*B* = 0.01, *SE* = 0.02, *t*(268) = 0.44, *p* = 0.66]; rumination [*B* = 0.01, *SE* = 0.01, *t*(268) = 0.17, *p* = 0.86]). 0 To summarise, shame acted as a perfect mediator in the relationship between self-compassion and eating pathology.

**Table 3:** The results of the mediator analyses, testing the roles of hypothesised factors in explaining the links of self-compassion with eating pathology and body image (intention to treat analyses—pooled results from 20 imputations, *N* = 274)

Step		B	*SE*	*t*	*p*
1	Self-compassion > eating pathology	− 0.54	0.14	− 4.04	0.001
2	Self-compassion > discrepancy perfectionism	− 4.68	0.56	8.42	0.001
	Self-compassion > standard perfectionism	− 0.16	0.40	0.41	0.68
	Self-compassion > rumination	− 14.67	2.67	5.49	0.001
	Self-compassion > shame	− 12.07	1.32	9.12	0.001
3	Discrepancy perfectionism > eating pathology	0.01	0.02	0.55	0.58
	Standard perfectionism > eating pathology	− 0.05	0.03	− 0.20	0.84
	Rumination > eating pathology	0.01	0.01	.18	0.86
	Shame > eating pathology	0.04	0.01	4.10	0.001
4	Self-compassion > discrepancy perfectionism > eating pathology	0.01	0.02	0.44	0.66
	Self-compassion > standard perfectionism > eating pathology	− 0.01	0.03	− 0.19	0.77
	Self-compassion > rumination > eating pathology	0.01	0.01	0.17	0.86
	Self-compassion > shame > eating pathology	0.04	0.01	3.93	0.001

Step		B	*SE*	*t*	*p*
1	Self-compassion > body dissatisfaction	− 10.22	1.78	5.76	0.001
2	Self-compassion > disc perfectionism	− 4.68	0.56	8.42	0.001
	Self-compassion > standard perfectionism	− 0.16	.40	0.41	0.68
	Self-compassion > rumination	− 14.67	2.67	5.49	0.001
	Self-compassion > shame	− 2.07	1.32	9.12	0.001
3	Discrepancy perfectionism > body dissatisfaction	0.68	0.25	2.74	0.05
	Standard perfectionism > body dissatisfaction	0.28	0.32	0.88	0.38
	Rumination > body dissatisfaction	0.08	0.09	0.97	0.34
	Shame > body dissatisfaction	0.37	0.12	3.05	0.05
4	Self-compassion > disc perfectionism > body dissatisfaction	0.59	0.28	2.10	0.05
	Self-compassion > standard perfectionism > body dissatisfaction	0.30	0.32	0.95	0.35
	Self-compassion > rumination > body dissatisfaction	0.08	0.09	0.98	0.33
	Self-compassion > shame > body dissatisfaction	0.33	0.15	2.25	0.05

#### Body image

##### Hypothesis 1

Higher levels of self-compassion were associated body image dissatisfaction over the full period of 6 months [*B* = –10.22, *SE* = 1.78, *t*(272) = 5.76, *p* < 0.001].

##### Hypothesis 2

The findings are as detailed above.

##### Hypothesis 3

At time 2, only higher levels of shame [*B* = 0.37, *SE* = 0.12, *t*(269) = 3.05, *p* < 0.05] and discrepancy perfectionism [*B* = 0.68, *SE* = 0.25, *t*(269) = 2.74, *p* < 0.05] 0 significantly predicted higher body image dissatisfaction at time 3. The remaining time 2 potential mediators were not related to body image dissatisfaction (standard perfectionism [*B* = 0.28, *SE* = 0.32, *t*(269) = 0.88, *p* = 0.38]; and rumination [*B* = 0.08, *SE* = 0.09, *t*(269) = 0.97, *p* = 0.34]).

##### Hypothesis 4

In the final mediational stage of the analysis, self-compassion [*B* =  − 2.19, SE = 2.68, *t*(268) = 0.82, *p* = 0.42] was no longer a significant predictor of body image after controlling for the mediating variables. Of the four potential mediators, shame [*B* = 0.33, *SE* = 0.15 *t*(268) = 2.25, *p* < 0.05] and discrepancy perfectionism [*B* = 0.59, *SE* = 0.28, *t*(268) = 2.10, *p* < 0.05] each played a significant role. The remainder did not mediate the self-compassion-body image dissatisfaction link (standard perfectionism [*B* = 0.30, *SE* = 0.32, *t*(268) = 0.95, *p* = 0.35]; rumination [*B* = 0.08, *SE* = 0.09, *t*(268) = 0.98, *p* = 0.33]). To summarise, shame and discrepancy perfectionism acted as perfect mediators in the link between self-compassion and body image dissatisfaction.

## Discussion

The aim of the present study was to investigate the factors that might link self-compassion with eating pathology and body dissatisfaction among women, using a longitudinal design over a period of 6 months in a community sample. Potential mediating factors were self-criticism, shame, rumination and perfectionism. Our findings demonstrate that shame is a perfect mediator of the link between self-compassion and eating pathology, while both shame and discrepancy perfectionism perfectly mediate the link between self-compassion and body dissatisfaction.

The pattern of bivariate associations reflected previous studies [e.g., 11, 12, 13, 14, 15]. In particular, the link between greater self-compassion and lower eating pathology and body dissatisfaction is consistent with conclusions from cross-sectional data in a recent meta-analysis [7]. These results are in line with a previous study, which reported that a mediating role of shame in the link between self-compassion and depression [15]. In particular, the mediating role of shame in the relationship between eating pathology/body dissatisfaction is similar to that found in a cross-sectional study (Turk et al., under review). Similarly, findings from a lab-based study showed that body shame mediated the relationship between self-compassion and anticipated disordered eating [40]. However, this is the first study to confirm such a model longitudinally, making the findings more likely to be meaningful. The fact that women who were self-compassionate had more positive body image appears to be because they feel less shame. This is in keeping with Kelly and Tasca’s (2016) finding that patients with an eating disorder experience lower levels of shame after a period of increased self-compassion during treatment [41]. However, it is also important to understand the mediating role of discrepancy perfectionism, which appears to reflect women who are more self-compassionate being less concerned about failing to get things right.

These results are in line with theories on the role of emotions/and or emotion regulation in eating pathology [42]. It can be hypothesised that self-compassionate individuals are more aware of their thoughts and emotions, and approach them with a non-judgmental attitude [6]. This lack of judgement might lead them to feel less shame. Therefore, when such individuals experience negative emotions, they do not engage in eating pathology behaviours to manage those emotional states.

This study has a number of limitations, which need to be addressed in future research. First, such research should sample more diverse participants in terms of gender, ethnicity, and clinical status (particularly those with eating disorders). Similarly, there is potential for bias due to the exclusion of non-English speakers, and future studies should investigate this model in non-English speaking samples. Second, more robust measures that do not rely on self-report would reduce the potential impact of bias in the participants’ responses. Third, there are multiple measures of perfectionism, which measure somewhat different constructs [41–45]. Therefore, future research will be needed to determine which conceptualisations and measures of perfectionism are most useful in understanding the impact of self-compassion. Fourth, considering possible biases in self-reported BMI [46], future research should measure weight and height objectively.

The results of the current study have potential implications for reducing eating pathology and negative body image in women, either in treatment or prevention work. Self-compassion interventions reduce eating pathology in both clinical and non-clinical populations [7, 47, 48], especially when implemented early in therapy [49]. These findings suggest that such self-compassion work should be used particularly where the individual experiences shame and/or where the individual fears getting things wrong, and that shame and discrepancy perfectionism should be assessed at the outset of treatment.

Taken as a whole, this longitudinal evaluation supports the role of shame as mediator between self-compassion and both eating pathology and body dissatisfaction. It also demonstrates a role for discrepancy perfectionism in explaining body image. Experimental studies (e.g., manipulating shame) would help us to establish a greater degree of accuracy in this matter.

## Conclusion

This longitudinal study contributes to our understanding of the role of self-compassion in terms of eating pathology and body image concerns, and particularly adds to the evidence that this relationship develops over time. Overall, the results highlight the importance of shame and perfectionism, emphasising their relevance during prevention and treatment. These findings indicate that such mechanisms should be considered when understanding the impact of such interventions for eating pathology and body concerns.

### What is already known on this subject?

The relationship between self-compassion and eating pathology/body image concerns is already established. However, there is a lack of research about mediators that could explain how self-compassion has its impact.

### What does this study add?

This study provides more definitive evidence that shame fully mediates self-compassion’s link to eating pathology and body image concerns over 6 months, and that discrepancy perfectionism also plays a role in the link between self-compassion and body dissatisfaction.

## Data Availability

The data that support the findings of this study are available on request from the corresponding author.
